# Editorial: The environment-animal-human web: a “One Health” view of toxicological risk analysis, volume II

**DOI:** 10.3389/fpubh.2023.1208636

**Published:** 2023-07-11

**Authors:** Chiara Frazzoli, Alberto Mantovani

**Affiliations:** ^1^Department of Cardiovascular, Endocrine-Metabolic Diseases and Ageing, National Institute of Health (ISS), Rome, Italy; ^2^Department of Food Safety, Nutrition and Veterinary Public Health, National Institute of Health (ISS), Rome, Italy

**Keywords:** antimicrobial resistance, education, research, nutrition, food safety, environment, prevention, risk analysis

The drive for development and modernization has come at considerable cost. The double burden of disease (co-existence of communicable and non-communicable or chronic diseases) is a reality, and risk factors due to chemicals and microorganisms in the environment are coming into sharper focus. With intensive environmental exploitation, climate-related health changes, dietary transition (including nutrition and safety), race to the modernity of products (formal/informal global market), and poor basic facilities (e.g., water and sanitation, WASH), the disease burden will continue to increase and evolve in the absence of strong preventive actions. A most interesting aspect of this process is the growing awareness that risk factors cannot be seen in isolation: nutritional imbalances, toxicant exposures, infectious agents and social inequity may synergize in many ways. The current “pandemics” of antimicrobial resistance is a telling example of interaction among factors previously considered as separate and independent: agro-farming practices, waste disposal, changes in biodiversity and animal ecology and even presence of toxic elements and pesticides, all build-up an important environmental component of the risk of antimicrobial resistance, which has still to be fully characterized.

Though many developing countries are setting up comprehensive food safety policies that, finally, include also toxic chemicals, the actual implementation of these policies is still weak. Efficient and effective health surveillance systems and prevention mechanisms struggle to grow, as well as risk assessment frameworks for environmental and food safety. The work at the interface between toxicology, microbiology and nutrition should engage in operational research and community-based approaches to truly boost One Health strategies in national health systems.

According to operational definition set by the quadripartite (WHO/FAO/WOAH/UNEP), One Health addresses health threats at the human-animal-environment interface based on collaboration, communication, and coordination across all sectors and disciplines, with the ultimate goal of achieving optimal health outcomes for people, animals and the environment. Hence, humanities (philosophy, ethics, history, comparative literature and religion), social science (psychology, sociology, anthropology, cultural studies, health geography) are also called to contribute developing and pushing the One Health approach in public health. This new concept of One Health, when operationalized, would be the best way to bring novel and fit-to-purpose scientific evidence on avoidable health risk factors in both production and consumption of healthy products and environmental health. For instance, food safety *from field to bowl* should increasingly cross-talk with food security and sustainability (e.g., food loss and waste, nutrition), community health (e.g., health education and empowerment, reproductive and mother-child health, and the salutogenic analysis of social determinants of health) and environmental health (e.g., emissions, biodiversity, changes in climate, urban and farming landscapes).

The papers contributing to the Research Topic provide interdisciplinary evidence and viewpoints in order to develop the new and emerging aspects of One Health. Attention is given to antimicrobial resistance as major issue calling for integrated efforts, as well as model field for One Health implementation. The papers concern One Health tools for risk assessment and surveillance (Aenishaenslin et al.; Haworth-Brockman et al.) and the identification of resistance reservoir in the farm ecosystem (Salerno et al.). The integration of food safety and nutritional security is considered in the emerging scenario of urban agriculture (Ebenso et al.). Last but not least, One Health can be pivotal for evidence-based priority setting, a topic area for the prevention of, and preparedness toward, food-related hazards and emerging threats (Zhao et al.).

Hence, the papers published in this Research Topic contribute to the conceptual and operational development of One Health. Strategic objectives of multidimensional work should pivot on information to overcome ideological limits, networking to face operational barriers (such as the insufficiency of interoperable data sets), research to improve technical level, advocacy to push policy/political action, moving resources to overcome economic constraints.

## Author contributions

All authors listed have made a substantial, direct, and intellectual contribution to the work and approved it for publication.

